# Conformational Analysis of the *Streptococcus pneumoniae* Hyaluronate Lyase and Characterization of Its Hyaluronan-specific Carbohydrate-binding Module*[Fn FN2]

**DOI:** 10.1074/jbc.M114.578435

**Published:** 2014-08-06

**Authors:** Michael D. L. Suits, Benjamin Pluvinage, Adrienne Law, Yan Liu, Angelina S. Palma, Wengang Chai, Ten Feizi, Alisdair B. Boraston

**Affiliations:** From the ‡ Department of Biochemistry and Microbiology, University of Victoria, Victoria, British Columbia V8W 3P6, Canada,; the § Glycosciences Laboratory, Imperial College London, Burlington Danes Building, Du Cane Road, London W12 0NN, United Kingdom, and; ¶ REQUIMTE, Department of Chemistry, Faculdade de Ciências e Tecnologia, Universidade Nova de Lisboa, 2829-516 Caparica, Portugal

**Keywords:** Carbohydrate, Carbohydrate Processing, Carbohydrate-binding Protein, Crystallography, Glycobiology, Hyaluronan, Small Angle X-ray Scattering (SAXS), Streptococcus, Hyaluronate Lyase

## Abstract

For a subset of pathogenic microorganisms, including *Streptococcus pneumoniae*, the recognition and degradation of host hyaluronan contributes to bacterial spreading through the extracellular matrix and enhancing access to host cell surfaces. The hyaluronate lyase (Hyl) presented on the surface of *S. pneumoniae* performs this role. Using glycan microarray screening, affinity electrophoresis, and isothermal titration calorimetry we show that the N-terminal module of Hyl is a hyaluronan-specific carbohydrate-binding module (CBM) and the founding member of CBM family 70. The 1.2 Å resolution x-ray crystal structure of CBM70 revealed it to have a β-sandwich fold, similar to other CBMs. The electrostatic properties of the binding site, which was identified by site-directed mutagenesis, are distinct from other CBMs and complementary to its acidic ligand, hyaluronan. Dynamic light scattering and solution small angle x-ray scattering revealed the full-length Hyl protein to exist as a monomer/dimer mixture in solution. Through a detailed analysis of the small angle x-ray scattering data, we report the pseudoatomic solution structures of the monomer and dimer forms of the full-length multimodular Hyl.

## Teaser

**Background:** Hyl from *Streptococcus pneumoniae* degrades host hyaluronan, an important polysaccharide component of the mammalian extracellular matrix.

**Results:** Hyl has a hyaluronan-specific carbohydrate-binding module, an extended conformation, and a propensity to dimerize.

**Conclusion:** Hyl both binds and degrades hyaluronan, which is facilitated by its cell surface presentation.

**Significance:** This is the first structural and functional characterization of a glycosaminoglycan-specific carbohydrate-binding module.

## Introduction

Hyaluronic acid, or hyaluronan, is a linear polysaccharide composed of repeating disaccharide units of d-glucuronic acid and d-*N*-acetylglucosamine linked by alternating β-1,4 and β-1,3 glycosidic bonds. As a principal component of the extracellular matrix, this comparatively simple, non-sulfated glycosaminoglycan is widely distributed throughout connective, epithelial, and neural tissues. Hyaluronan plays an integral role in mediating a multitude of interactions in key biological events, such as wound healing, fertilization, cancer cell growth and metastasis, immune signal propagation, and as a shock-absorbing component of synovial fluid (reviewed by Jiang *et al.* (1)).

Because of its ubiquitous presence as an extracellular matrix component, hyaluronan recognition and processing is an important intersection between various pathogens and animal hosts, as is the case with *Streptococcus pneumoniae*. This bacterium is a common commensal that colonizes the nasopharynx of ∼40% of humans without causing any adverse symptoms. *S. pneumoniae*, however, also possesses a potent capability for invasive infection, causing a broad spectrum of diseases ranging from common ear infections (otitis media) to more lethal infections, such as acute respiratory disease, bacteremia, and meningitis (2). The interaction of *S. pneumoniae* with its host is highly complex, involving a number of elements (2), but one of the earliest known factors is a hyaluronate-degrading enzyme (3, 4), whose activity is highly correlated with invasive *S. pneumoniae* infections (5, 6). Subsequent identification of the *hyl* gene encoding this enzyme (7), Hyl, has promoted detailed study of its contribution to virulence. In animal models, the direct contribution of Hyl to virulence is not significant; however, the production of Hyl by the bacterium has a strong potentiating effect on the critical virulence factor pneumolysin, a pore-forming cytotoxin that binds to host cholesterol (8, 9). This potentiating effect is consistent with Hyl playing a role in breaking the integrity of the extracellular matrix barrier, thereby providing improved access of the bacterium and pneumolysin to the host cell surface. In addition to the “spreading” action of Hyl, this protein provides the bacterium with the capability of using hyaluronan as a carbon source, which is linked to colonization and persistence of the bacterium (10). Given the capability of Hyl to degrade the hyaluronan capsules of other potentially competing bacteria, such as *Streptococcus pyogenes*, Hyl may also play a niche-specific role in bacterial warfare (10).

Hyl is a 1066-amino acid protein that is anchored to the bacterial cell surface through a sortase-mediated process, although soluble Hyl can also be found (11). A catalytically active fragment of Hyl comprising amino acids ∼300–1020 has been well characterized both structurally and functionally (for examples, see Refs. 11 and 12). By amino acid sequence identity, this region of Hyl classifies it as a family 8 polysaccharide lyase (PL8)^5^ (13). The PL8 domain of Hyl comprises a (α/α)_6_ barrel (α-domain) fused to an anti-parallel β-sheet (β-domain) that caps the active site in the (α/α)_6_ barrel. This catalytically active PL8 domain enables Hyl to cleave hyaluronan using a β-elimination reaction mechanism, thus releasing a disaccharide product with a 4,5-unsaturated non-reducing end d-glucuronic acid.

The N-terminal region of Hyl remains uncharacterized. Through the results of bioinformatics approaches, the amino acids from roughly position 50 to 210 in Hyl were proposed to comprise a carbohydrate-binding module (CBM) (14). CBMs are common ancillary modules in carbohydrate-active enzymes, where they function to target and hold the parent enzyme in proximity to substrates, thereby enhancing enzyme efficiency (15). Like carbohydrate-active enzyme catalytic modules, CBMs are classified into amino acid sequence-based families (13). The binding specificity of CBMs most commonly matches that of the appended catalytic module (15). Indeed, Rigden and Jedrzejas (14) hypothesized that the N-terminal domain of Hyl is a hyaluronan-binding CBM that is similar in structure to the β-glycan binding family 4 CBMs.

Although the structures of several hyaluronan-specific adherins (hyaladherins) have been determined, they are all of eukaryotic origin and are involved in normal “self-recognition” of hyaluronan in the extracellular matrix (16–18). At present, there is comparatively little information regarding the molecular recognition of hyaluronan by pathogenic microbes. To address this knowledge gap and test the hypothesis that the N-terminal module of Hyl is a hyaluronan-specific CBM, we examined the structural and functional properties of this module using a combination of approaches, including glycan microarray, isothermal titration calorimetry, x-ray crystallography, and site-directed mutagenesis. Furthermore, we studied full-length Hyl using solution small angle x-ray scattering (SAXS) to reveal the first complete experimentally based model of the Hyl conformation. The results reveal that Hyl possesses an N-terminal hyaluronan-binding CBM that is the founding member of CBM family 70. The full-length protein adopts an extended conformation and dimerizes in solution. The SAXS-based model of Hyl, interpreted on the basis of its surface presentation, suggests a conformation optimized for the interaction of the CBM with hyaluronan present in the extracellular matrix.

## EXPERIMENTAL PROCEDURES

### 

#### 

##### Cloning

The oligonucleotide PCR primers used for the amplification of the following constructs from genomic DNA are presented in Table 1. Gene fragments encoding SpCBM70 (residues Lys^53^–Ser^212^) and a construct including the CBM, middle, and PL8 domains of Hyl (residues Lys^53^–Lys^1006^; hereafter referred to as simply Hyl) were cloned via standard PCR, restriction endonuclease, and ligation procedures into the plasmid pET28 (EMD Millipore), using NdeI and HindIII sites to yield *p*SpCBM70 and *p*SpHyl expression vectors, respectively. The gene fragment encoding the PL8 catalytic domain (residues Lys^288^–Gln^1009^) was cloned into pET28, using the In-Fusion HD cloning procedure (Clontech). All of the constructs above encoded the desired polypeptides fused to an N-terminal six-histidine tag by a thrombin cleavage site. An additional SpCBM70 construct (SpCBM70-C) encompassing residues Asn^54^–Ser^212^ was cloned into pET28 using NotI and NcoI sites to yield *p*SpCBM70-C, which encoded SpCBM70 with a C-terminal six-histidine tag. SpCBM70-C mutants (W82A, R112A, H116A, K143A, R145A, and K185A) were generated from the SpCBM70-C construct using standard PCR mutagenesis procedures.

**TABLE 1:** **Oligonucleotide primers**

Oligonucleotide	Primer sequence (5′–3′)	Used to amplify and clone
N-CBM-for	GGCATATGCCATGGAAAATTTAGTTGAAAATGGTG	Hyl, SpCBM70
N-CBM-rev	TTTTGAAGCTTTTAAGAAAGCTGGTCTGC	SpCBM70
C-CBM-for	GTGGTGCTCGAGTTAGCTTTTGATGAACGAAATG	SpCBM70C, CBM mutants
C-CBM-rev	CCAAGCTTGCGGCCGCAGAAAGCTGGTCTGC	SpCBM70C, CBM mutants
Hyl-rev	TTTTGAAGCTTTTACTTTTTAAAGACTTCC	Hyl
W82A-for	CAGGGGTGGTCAGCTGCGGTAGACCAGAAGAA	W82A
W82A-rev	ATTCTTCTGGTCTACCGCAGCTGACCACCCCTG	W82A
R112A-for	AGCCATGAGAAATTAGCGGCAGCGCTTCACCGT	R112A
R112A-rev	ACGGTGAAGCGCTGCCGCAATTTTCTCATGGCT	R112A
H116A-for	TTAAGGGCAGCGCTTGCGCGTATGGTTCCTATT	H116A
H116A-rev	AATAGGAACCATACGCGCAAGCGCTGCCCTTAA	H116A
K143A-for	AAAATCGGGATTGCCGCGGTTCGTATCATTGAG	K143A
K143A-rev	CTCAATGATACGAACCGCGGCAATCCCGATTTT	K143A
R145A-for	GGGATTGCCAAAGTTGCGATCATTGAGGAAAGT	R145A
R145A-rev	ACTTTCCTCAATGATCGCAACTTTGGCAATCCC	R145A
K185A-for	GATGTTGATAAAATCGCGCTGGAGTTATTCTAT	K185A
K185A-rev	ATAGAATAACTCCAGCGCGATTTTATCAACATC	K185A
PL8-for	CAGCCATATGGCTAGCAAGGATGCATACACAGACC	PL8
PL8-rev	GGTGGTGGTGCTCGAGTTATTGCTCTAGCTTTTTAAAGAC	PL8

##### Protein Production and Purification

In all cases, recombinant proteins were produced via the autoinduction method (19), using shaking 1-liter cultures supplemented with kanamycin (50 μg/ml) for 36 h at 37 °C, reducing the temperature to 16 °C, and continuing growth for an additional 48 h. Cells were harvested by centrifugation and disrupted by a lysozyme/chemical lysis method using the sucrose-deoxycholate procedure, all buffered with PBS (50 mm Na_2_HPO_4_, 300 mm NaCl, pH 7.5) throughout. Briefly, pelleted cells were resuspended in 25 ml of 25% sucrose-PBS. 10 mg of lysozyme was added while stirring, followed by a 10-min incubation at 20 °C. 50 ml of a mixture of 1% deoxycholate, 1% Triton X-100-PBS was subsequently added at 4 °C, and the mixture was stirred for 10 min. DNase was added to 2 μg/ml, and MgCl_2_ was added to 5 mm, and the mixture was stirred at 20 °C for 15 min. Clarified lysates were purified via immobilized metal affinity chromatography and anion exchange chromatography. Proteins in 20 mm Tris-HCl (pH 8.0) and 100 mm NaCl were concentrated using a stirred ultrafiltration cell with a 10,000 molecular weight cut-off filter. Protein concentrations were determined by measuring absorbance at 280 nm (*A*_280_) and using calculated molar extinction coefficients of 26,470 and 162,040 cm/m for SpCBM70W82A and Hyl, respectively, 127,909 cm/m for PL8, and 31970 cm/m for SpCBM70, SpCBM70-C, and the remaining SpCBM70 mutants (20).

##### Glycan Microarray Analyses

The glycan binding specificity of SpCBM70 was first analyzed using the Glycosciences Screening Array Set 32–39, comprising 492 lipid-linked oligosaccharide probes of neoglycolipids (NGLs) or glycosylceramides (see supplemental Table 1) (21).

Additional analysis was performed using a focused glycosaminoglycan (GAG) microarray of 25 GAG oligosaccharide probes prepared as NGLs (supplemental Table 2). The GAG oligosaccharides were obtained by partial depolymerization of the polysaccharides using lyases and size-fractionated by gel filtration chromatography as described (22). The oligosaccharide fractions were analyzed by negative ion electrospray ionization mass spectrometry after conversion into their ammonium salts for determination of the chain lengths of the major components in the fractions. For arraying, the oligosaccharides were converted into NGLs by reductive amination for chondroitin sulfate and hyaluronic acid and by oxime ligation for heparan sulfate and heparin oligosaccharides (23, 24). The NGL probes were purified, analyzed, and quantified before robotically printing onto nitrocellulose-coated 16-pad glass slides at 2 and 7 fmol/spot in duplicate.

For microarray binding analyses, the arrayed slides were blocked with 3% (w/v) bovine serum albumin (BSA; Sigma-Aldrich) in 5 mm HEPES buffer, pH 7.4, containing 150 mm NaCl, 5 mm CaCl_2_ and overlaid for 1.5 h at ambient temperature with SpCBM70 precomplexed with mouse monoclonal anti-polyhistidine (Ab1) and biotinylated anti-mouse IgG antibodies (Ab2) (both from Sigma) in a ratio of 1:3:3 (by weight). The complex was prepared by preincubating Ab1 with Ab2 for 15 min at ambient temperature, followed by the addition of SpCBM70 and incubation for a further 15 min. The complex was diluted with 1% BSA (w/v) to give a final SpCBM70 concentration of 10 μg/ml for overlaying. Carbohydrate binding was detected with Alexa Fluor 647-labeled streptavidin. Data analysis was performed with dedicated microarray software (25).

##### Isothermal Titration Calorimetry

Isothermal titration calorimetry (ITC) was performed using a VP-ITC (MicroCal, Northampton, MA) at 25 °C in each case. Due to the viscosity associated with concentrated hyaluronan solutions, concentrated SpCBM70 was titrated into hyaluronan solutions. Briefly, SpCBM70 was extensively dialyzed against 25 mm Tris-HCl (pH 7.5) and 50 mm NaCl and concentrated as described above. Dialysate was retained and subsequently used for dissolution or dilution of reagents. In each case, protein and carbohydrate solutions were degassed immediately prior to use. Samples of 2.65 mm solution of SpCBM70 were titrated into a solution of 0.142 mg/ml hyaluronan (corresponding to an equivalent concentration of 125 μm of a 6-mer fragment). Heat of dilution corrected data were fit to a one-site binding model to yield the association constant (*K_a_*), change in enthalpy (Δ*H*), and stoichiometry (*n*). The change in Gibb's free energy (Δ*G*) and the change in entropy (Δ*S*) were calculated from these values.

Purified fragments of hyaluronan of defined length (Oligo-4, nanoHA^TM^_5_, Oligo-6, and nanoHA^TM^_7_, all from Sigma-Aldrich) were titrated into SpCBM70 (>150 μm) under conditions maintained such that *C* values were in excess of 5. Heat of dilution corrected data were fit to a one-site binding model to yield the binding parameters.

##### Affinity Gel Electrophoresis

The interaction of SpCBM70-C and mutants with hyaluronan was analyzed by affinity electrophoresis. 9% (w/v) polyacrylamide gels (pH 8.8), were polymerized with varying concentrations of hyaluronan (Sigma-Aldrich) ranging from 0 to 0.045% final concentration. SpCBM70-C, the six mutants, and three non-interacting control proteins were all run for 3.5 h at a constant voltage of 150 V in an ice bath. Gels were stained with Coomassie Brilliant Blue R-250. Affinities were determined as described previously (26) and expressed as relative affinities with the assumption of identical stoichiometries for all of the proteins binding to the polysaccharide.

##### SpCBM70 Crystallization and Structure Determination

SpCBM70-C supplemented with a 2-fold molar excess of hyaluronan tetrasaccharide crystallized at a concentration of 20 mg/ml by the sitting drop vapor diffusion method by mixing equal amounts of the protein solution with a mother liquor consisting of 18% (w/v) polyethylene glycol 3,350 and 0.1 m sodium acetate (pH 5.5) at 292 K. Hexagonal plate-shaped crystals developed after ∼1 day and were subsequently cryoprotected with 25% ethylene glycol prior to diffraction experiments. A bromide derivative data set was obtained by soaking an SpCBM70 crystal for 5 min in crystallization solution supplemented with 1 m NaBr prior to cryoprotection and freezing.

Diffraction data from the bromide derivative and native crystals were both collected at the beamline 08B1-1 at the Canadian Macromolecular Crystallography Facility (Canadian Light Source) using 0.5º oscillations. Data sets were processed with XDS and scaled with Scala (27, 28). Bromide sites for phasing were identified with ShelxC/D, and initial phasing was performed with ShelxE (29). The resulting phases were of sufficient quality for a nearly complete model to be automatically built with ARP/wARP (30). This model was subsequently used as a starting model for the native structure. Model building was performed with Coot, and refinement was performed with Refmac (31, 32). The final refinement incorporated the addition of hydrogen in the riding positions and anisotropic *B*-factors. Structure validation was performed with Molprobity (33). Data collection, processing, and refinement statistics are presented in Table 2. Figures were generated using PyMOL.

**TABLE 2:** **X-ray data collection, processing, and SpCBM70 model refinement statistics** The coordinates of SpCBM70 are available under the PDB accession code 4D0Q.

	Bromide peak	Native
**Data collection statistics**		
Wavelength	0.91951	0.95371
Beamline	CLS 08B1-1	CLS 08B1-1
Space group	*P*2_1_	*P*2_1_
Resolution	34.0-1.30 (1.37-1.30)*^a^*	35.3-1.20 (1.26-1.20)
Cell dimension	34.4, 61.1, 35.3	34.4, 60.8, 35.5
α, β, γ (Å)	90.0, 94.0, 90.0	90.0, 95.5, 90.0
*R*_merge_	0.059 (0.435)	0.041 (0.393)
Completeness (%)	98.8 (97.6)	99.4 (96.2)
〈*I*/σ*I*〉	22.2 (4.6)	26.7 (4.5)
Redundancy	7.6 (7.6)	7.3 (6.4)
Total reflections	335,981	370,567
Unique reflections	35,392	45,171

**Refinement statistics**		
*R*_work_/*R*_free_ (%)		12.7/15.9
Root mean square deviations		
Bond lengths (Å)		0.016
Bond angles (degrees)		1.630
Average *B*-factors (Å^2^)		
Protein molecule		13.7
Solvent molecules		34.3
No. of atoms		
Protein atoms		2668
Solvent atoms		307
Ramachandran statistics		
Most favored (%)		97.3 (145)
Additional allowed (%)		2.7 (4)
Disallowed (%)		0 (0)

*^a^* Values in parentheses represent data for the highest resolution shell.

##### Small Angle X-ray Scattering

After a purification step of size exclusion chromatography and filtering through a 0.22-μm filter, Hyl samples were examined by dynamic light scattering (DLS). DLS data were collected at 25 °C in triplicate, averaging 15 acquisitions of 5 s using a DynaPro plate reader (Wyatt Technology, Santa Barbara, CA). The data were then analyzed using the Dynamics version 7.1 software. The molecular weight was determined based on a globular protein model.

Synchrotron x-ray scattering data from purified solutions of Hyl in 50 mm Tris-HCl (pH 8.0) and 500 mm NaCl at concentrations ranging from 1.0 to 10.8 mg/ml were collected at beamline 4-2 at the Stanford Synchrotron Radiation Lightsource (SSRL) using a MarCCD 165 detector. A solution (1 mg/ml) of bovine serum albumin was measured as a reference and for calibration purposes. Diffraction data were measured with an exposure time of 1 min at 288 K with a wavelength of 1.50 Å. The sample-to-detector distance was set at 2.5 m, leading to scattering vectors *q* (defined as *q* = 4π/λsinθ, where 2θ is the scattering angle) ranging from 0.06 to 0.5 Å^−1^. SAXS data processing and determination of the radii of gyration (*R_g_*), and maximum particle size (*D*_max_) were performed in accordance with procedures described previously (34). The *ab initio* low resolution envelopes of Hyl were generated with DAMMIF (35), using 10 independent runs with no shape constraints. The solutions were then aligned and averaged using the DAMAVER suite of programs (36), which also computes the NSD values for the groups of *ab initio* models (37).

The rigid body modeling of Hyl was performed with SASREF7MX (38) using the structures of the three domains comprising SpCBM70 (residues Lys^53^–Ser^212^), the linker domain (residues Glu^213^–Val^287^, modeled based on the linker portion of the *Streptococcus agalactiae* hyaluronate lyase, PDB entry 1I8Q), and PL8 catalytic domain (residues Lys^288^–Lys^1006^, PDB entry 1LXK) (39) as separate rigid bodies. Maximum interdomain distances approximating the short amino acid linkers between N and C termini were used as constraints, and P2 symmetry was used to model the monomer and dimer mixture observed in solution. The volumes of associated fractions were determined using SASREF7MX as well as the OLIGOMER program (40, 41).

## RESULTS

### 

#### 

##### The N Terminus of Hyl Is a Hyaluronan-binding Module

The hyaluronan-degrading activities of the isolated PL8 domain of Hyl are well documented, both biochemically and structurally (11, 12). The hyaluronan binding activity of the putative N-terminal CBM, however, is only hypothetical. To examine this, we cloned the gene fragment encoding the putative CBM, here called SpCBM70, and recombinantly produced this independent module in *Escherichia coli* and purified it for characterization.

**FIGURE 1.**
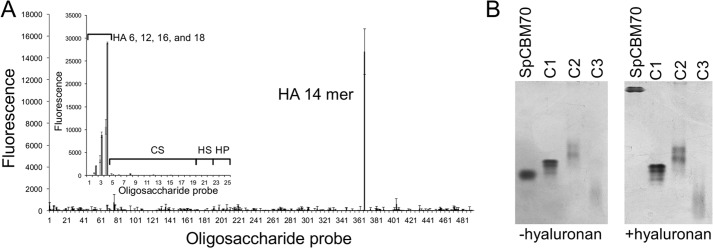
**Analyses of the glycan binding properties of SpCBM70.** *A*, microarray analysis of SpCBM70 binding specificity. The screening glycan microarray of 492 lipid-linked probes, NGLs, and glycosylceramides (>370 mammalian type) is the Glycosciences Array Set 32–39 described previously (21) (supplemental Table 1). Binding signals are expressed as numerical scores, means of fluorescence intensities of duplicate values recorded at 5 fmol oligosaccharide probe/spot. The *error bars* represent half of the difference between the two values. The *inset* shows microarray analyses with 25 lipid-linked GAG oligosaccharide probes. Numerical scores of the binding signals are means of duplicate spots (with *error bars*) of oligosaccharide probes printed at 2 (*light gray bars*) and 7 (*dark gray bars*) fmol/spot on nitrocellulose-coated glass slides. *HA*, *CS*, *HS*, and *HP*, hyaluronan, chondroitin sulfate, heparin sulfate, and heparin fragments, respectively. *B*, native polyacrylamide gel electrophoresis analysis of SpCBM70 in the absence of hyaluronan (−*hyaluronan*) and with hyaluronan present in the gel at a final concentration of 0.045% (w/v) (+*hyaluronan*). *C1*, *C2*, and *C3*, the migration of three non-interacting control proteins.

We initially screened SpCBM70 for carbohydrate binding using NGL arrays representing 492 distinct potential glycan receptors. SpCBM70 interacted only with a single glycan on the array, a 14-mer fragment of hyaluronan (Fig. 1*A*). To examine further its specificity, we used a focused GAG oligosaccharide microarray that comprises NGL probes derived from size-defined oligosaccharide fractions (ranging from 4-mers to 18-mers) of different types of GAG polysaccharides: hyaluronan; chondroitin sulfates CSA, CSB, and CSC; heparan sulfate; and heparin. Among these were 6-, 12-, 16-, and 18-mer fragments of hyaluronan. Substantial binding of SpCBM70 was observed to 12-mer and larger fragments of hyaluronan (Fig. 1*A*, *inset*). Binding to hyaluronan was further confirmed by qualitative affinity electrophoresis, which revealed a strong reduction in the mobility of SpCBM70 in gels polymerized with hyaluronan (Fig. 1*B*).

Using ITC, we quantified the binding of SpCBM70 to hyaluronan and hyaluronan fragments (Table 3). SpCBM70 displayed binding to a 4-mer fragment of hyaluronan; however, the interaction was too weak to quantify by ITC and is generally consistent with the lack of binding to this oligosaccharide on the glycan microarrays. The binding of SpCBM70 to longer fragments (5–7-mer) displayed a 1:1 stoichiometry and increasing affinity with increasing fragment length, the highest affinity being for the 7-mer at 3.5 ± 0.2 × 10^5^ m^−1^. The enthalpic contribution to binding decreased with chain length, whereas the entropic contribution increased considerably with favorable entropic contributions to binding for the 6- and 7-mer fragments. The favorable changes in Δ*H* and Δ*S* for binding to the 6- and 7-mer hyaluronan fragments are unusual for CBM carbohydrate interactions, which usually display favorable Δ*H* values with partially offsetting unfavorable Δ*S* values (15).

**TABLE 3:** **Isothermal titration calorimetry analysis of the SpCBM70 interaction with hyaluronan and hyaluronan fragments**

Carbohydrate	*n*	*K_a_*	Δ*H*	Δ*S*	Δ*G*
		*m*^−*1*^	*kcal/mol*	*cal/mol/K*	*kcal/mol*
Hyaluronan 4-mer		<5 × 10^3^			
Hyaluronan 5-mer	1.12 ± 0.00	0.7 ± 0.1 × 10^5^	−7.7 ± 0.3	−8.2 ± 1.1	−6.6 ± 0.1
Hyaluronan 6-mer	1.07 ± 0.06	1.4 ± 0.1 × 10^5^	−2.6 ± 0.1	14.8 ± 0.8	−7.0 ± 0.1
Hyaluronan 7-mer	0.96 ± 0.01	3.5 ± 0.2 × 10^5^	−2.4 ± 0.1	17.3 ± 0.5	−7.6 ± 0.1
Hyaluronan*^a^*	0.86 ± 0.02	1.3 ± 0.1 × 10^5^	−12.9 ± 1.0	−20.0 ± 3.4	−7.0 ± 0.1

*^a^* Polysaccharide concentration was calculated based on the molecular weight of a 6-mer fragment.

The interaction of SpCBM70 with hyaluronan was analyzed with ITC by titrating the CBM into a solution of the dissolved polysaccharide (Table 3). The concentration of the hyaluronan was treated as molar equivalents of a 6-mer, and this resulted in an *n* value of 0.86. This corresponds to one CBM binding to every ∼7-mer block on the polysaccharide, thus suggesting the potential for quite close packing of CBMs on hyaluronan at maximum loading. The affinity was similar to that found for the 6- and 7-mer hyaluronan fragments; however, the Δ*H* and Δ*S* values were markedly different and more consistent with what is typically seen for CBM carbohydrate interactions. The increased affinity for longer hyaluronan fragments combined with an increased number of binding sites may explain why only longer hyaluronan oligosaccharides were detected in the microarray analysis.

SpCBM70, therefore, is indeed a functional CBM, the founding member of CBM family 70, and represents the first CBM known to specifically interact with a glycosaminoglycan. Modules with significant amino acid sequence identity to SpCBM70, and therefore additional members of CBM family 70, are found predominantly in other streptococcal species. The amino acid sequence identity of these other putative CBM70s with SpCBM70 is as low as 30%. However, the observation that they are found in proteins annotated as hyaluronidases or hyaluronate lyases suggests that specificity for hyaluronan may predominate in CBM family 70.

##### Properties of the Hyl CBM Hyaluronan Binding Site

**FIGURE 2.**
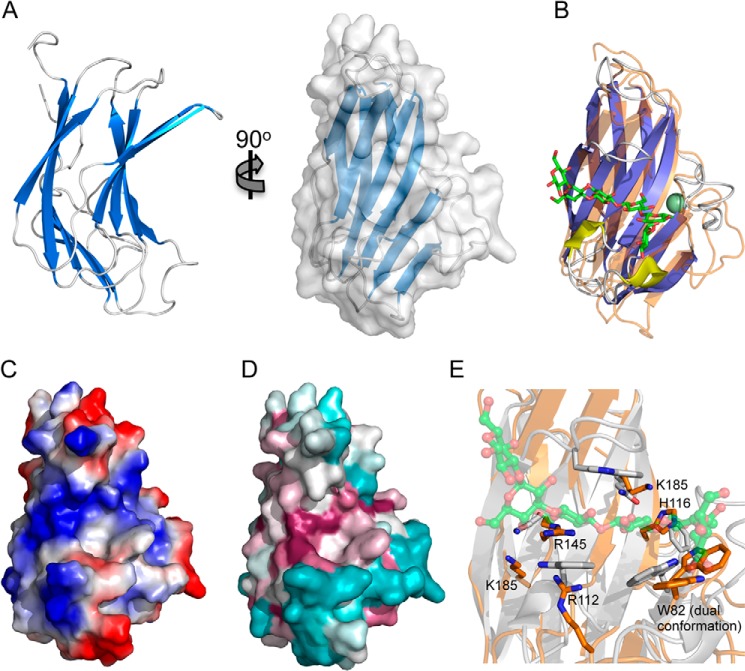
**The structure of SpCBM70.** *A*, schematic representation of the 1.2 Å resolution structure of SpCBM70 shown from two angles and with the solvent-accessible surface shown in *transparent gray* (*right*). *B*, a structural overlap of SpCBM70 (*transparent orange*) with TmCBM4–2 (*blue*/*yellow*) from *Thermotoga maritima* (PDB entry 1GUI). The β-1,3-glucohexaose molecule bound to TmCBM4–2 is shown as *green sticks*, and the bound calcium is shown as a *green sphere. C*, the solvent-accessible surface of SpCBM70 (shown from the same angle as in *B*) *colored* by electrostatic potential. *D*, the solvent-accessible surface of SpCBM70 (shown from the same angle as in *B*) *colored* by conservation of the residues among similar modules from *Streptococci* (generated using ConSurf (54)). *E*, detailed view of the overlap of the putative SpCBM70 hyaluronan binding site (*orange*) with the β-1,3-glucohexaose binding site in TmCBM4-2 (*gray*). Residues targeted for mutagenesis in SpCBM70 are shown as *sticks* and *labeled*.

Because it is the first hyaluronan-specific CBM we sought to provide insight into the molecular basis of hyaluronan recognition through structural studies of SpCBM70. The CBM crystallized in space group P2_1_ with one protein molecule in the asymmetric unit. An initial model of the protein was built using phases provided by a bromide-optimized single anomalous dispersion experiment, and data were collected to 1.3 Å resolution. The final model was built and refined using a native data set collected to 1.2 Å resolution and comprises 161 residues. The polypeptide adopts a β-sandwich fold of opposing 5-stranded antiparallel β-sheets with a jelly roll topology (Fig. 2*A*). The sandwich is slightly bowed, creating a groove that spans the β-sheet on the concave surface. Due to the common nature of this fold, SpCBM70 displays similarity to a large number of proteins; however, the most functionally relevant proteins with which SpCBM70 displays structural identity are the β-sandwich CBMs. Although they have insignificant amino acid sequence identity with SpCBM70, the most structurally similar CBMs are those in family 4, as typified by the β-1,3-glucan binding TmCBM4-2 from *Thermotoga maritima* (PDB entry 1GUI, root-mean-square-deviation of 1.98 Å as measured by secondary structure matching over 126 matched Cαs (42)) (Fig. 2*B*). Despite conservation of the overall fold, the metal-binding site found in the family 4 CBMs was not found in SpCBM70.

Notably, the carbohydrate-binding site of the CBM4s, which is invariably present on the concave surface of one β-sheet, is in the same general location as the groove on the surface of SpCBM70. This particular surface of SpCBM70 possesses both a distinct positive charge and a high degree of conservation with other sequences that fall into CBM family 70 (Fig. 2, *C* and *D*). Furthermore, a more detailed comparison of this surface region in SpCBM70 with the binding site of TmCBM4-2 reveals the conservation of a solvent-exposed surface tryptophan that in TmCBM4-2 is known to be critical for sugar binding (Fig. 2*E*) (42, 43). The complementarity of the electrostatic properties of this groove with hyaluronan, the conservation of surface residues among family CBM70, and the specific conservation of functionally important residues with CBM4 suggest that this site on SpCBM70 is the hyaluronan-binding site. Indeed, based on homology modeling, this site on SpCBM70 was also proposed by Rigden and Jedrzejas (14) as a putative binding site.

Because we were unable to generate a structure of SpCBM70 in complex with a fragment of hyaluronan, to provide additional evidence for the location of the binding site, we mutated the solvent-exposed tryptophan as well as basic residues in the putative binding groove (Fig. 2*E*) to alanine and assessed the binding of these mutants to hyaluronan by quantitative affinity electrophoresis. The mutations R112A and R145A abrogated binding, whereas the mutations W82A and K185A reduced binding by ∼50-fold (Table 4). The mutations H116A and K143A had more modest effects on affinity, reducing binding by ∼10-fold. These results conclusively identify the hyaluronan-binding site of SpCBM70 as residing in the groove that is conserved with family 4 CBMs. Furthermore, the results suggest that the recognition of hyaluronan involves the aromatic amino acid side chain-carbohydrate interactions that are typical of protein-carbohydrate interactions and ubiquitous to CBMs but also potentially electrostatic interactions, which are probably common in protein-GAG interactions but generally rare in protein-carbohydrate interactions.

**TABLE 4:** **Characterization of SpCBM70 and mutants binding to hyaluronan by affinity electrophoresis**

SpCBM70	Relative *K_d_*
WT	1*^a^*
W82A	54
R112A	NB*^b^*
H116A	8
R145A	NB
K143A	14
K185A	55

*^a^* The *K_d_* determined by affinity electrophoresis using a 7-mer binding site, as estimated from the ITC data, was 7.2 ± 0.8 μm, which corresponds to a *K_a_* of 1.4 × 10^5^ m^−1^.

*^b^* NB, no binding.

##### The Solution Structure of Hyl

Due to their structural irregularity, large multimodular enzymes, such as Hyl, typically resist crystallization, resulting in limited structural data relating the individual functions of composite modules to the overall conformation of the protein. In the case of Hyl, the evolving model of Hyl architecture is that it possesses hyaluronan-binding activity through its N-terminal CBM70 and hyaluronan-degrading activity in its C-terminal PL8 domain. How these modular functions are spatially coordinated is unknown, and given the surface presentation of the protein and its involvement in the bacterium-host interaction, understanding the function of Hyl is of considerable interest.

**FIGURE 3.**
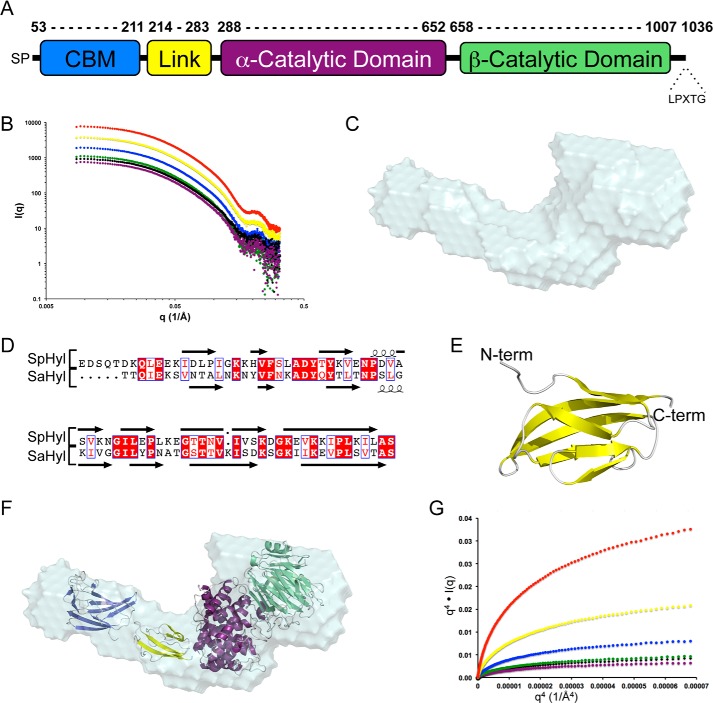
**Modular properties and initial SAXS analysis of full-length Hyl.** *A*, schematic of the modular architecture of Hyl. The α- and β-catalytic domains constitute the PL8 domain. *B*, SAXS scattering profiles of Hyl, protein concentrations: 10.80 (*red*), 5.40 (*yellow*), 2.70 (*blue*), 1.50 (*green*), 1.35 (*black*), and 1.00 mg/ml (*purple*). *C*, DAMAVER averaged shape of 10 independent DAMMIF-generated envelopes of Hyl using the data collected on the 1.0 mg/ml sample. *D*, alignment of the linker module from Hyl with the similar module from the *Streptococcus agalactiae* Hyl (*SaHyl*). *E*, Phyre2-generated model using the structure of the SaHyl linker domain as a template. *F*, the composite modules of Hyl (CBM (*blue*), linker (*yellow*), α-catalytic domain (*purple*), and β-catalytic domain (*green*)) manually placed into the DAMMIF envelope. *G*, Porod-Debye plots of the SAXS data for Hyl. *A*, *q*^4^·I(*q*) *versus q*^4^ plot for Hyl at 10.80 (*red*), 5.40 (*yellow*), 2.70 (*blue*), 2.4 (*green*), 1.35 (*black*), and 1.00 mg/ml (*purple*).

Despite extensive attempts, we were unable to crystallize full-length Hyl, so we addressed the issue of determining a full-length structure of this protein by an alternate approach. A Hyl construct comprising residues 53–1007 (Fig. 3*A*) and thus lacking the N-terminal secretion signal peptide and C-terminal cell-wall anchoring motif was purified by immobilized metal affinity chromatography and a final size exclusion chromatography step. Identical concentrations of pure Hyl (1.0–10.8 mg/ml) were examined by DLS and SAXS to examine the solution conformation of the full-length protein. Analysis by DLS revealed what appeared to be a single protein population comprising >99.8% of the sample by mass with a molecular mass of ∼150 kDa, a radius of ∼50 Å, and a polydispersity of ∼15–20% (Table 5). Consistent with the DLS results, the scattering profiles obtained for a range of Hyl concentrations were characteristic of monodisperse samples and indicated that increasing the protein concentrations does not result in significant aggregation (Fig. 3*B*). The molecular mass and radius of *R_g_* of ∼145 kDa and ∼47 Å, respectively, were in good agreement with the values determined by DLS, and the average particle density was ∼0.88 g/cm^3^ (Table 6).

**TABLE 5:** **Characterization of Hyl and its PL8 catalytic domain in solution by dynamic light scattering**

Concentration	Radius	Polydispersity	Mass	Percentage of mass	Percentage of associated fraction
*mg/ml*	Å	%	*kDa*	%	%
**Hyl**					
10.8	52.4 ± 1.9	14.7 ± 3.3	163 ± 13	99.9	43.0
5.4	51.3 ± 1.1	20.5 ± 6.0	154 ± 8	99.8	36.4
2.7	50.8 ± 0.8	16.9 ± 4.7	151 ± 6	100.0	33.5
1.5	49.7 ± 1.1	24.5 ± 4.8	143 ± 7	99.9	27.4
1.35	51.0 ± 1.5	14.9 ± 2.3	153 ± 11	99.8	34.7
1.0	50.8 ± 1.8	22.7 ± 5.3	151 ± 13	100.0	33.5

**PL8**					
10.9	45.8 ± 2.0	16.0 ± 7.2	118 ± 11	100.0	39.1
5.5	43.5 ± 1.7	14.4 ± 7.9	105 ± 10	100.0	24.3
2.7	42.9 ± 1.7	17.4 ± 4.4	102 ± 10	100.0	20.8
1.4	42.0 ± 0.8	14.9 ± 3.7	97 ± 5	100.0	15.6

**TABLE 6:** **Characterization of Hyl by small angle x-ray scattering**

Concentration	Molecular mass*^a^*	*R_g_* (Guinier)	*R_g_* (GNOM)	*D*_max_	Porod volume	Particle density*^b^*	*Ab initio* modeling	
							χ (DAMMIF)*^c^*	NSD*^d^*
*mg/ml*	*kDa*	Å	Å	Å	Å*^3^*	*g/cm^3^*		
**Hyl**	110.6*^e^*							
10.80	146.2	48.2 ± 1.0	49.5	170.5	211,709	0.87	1.93 ± 0.23	0.66 ± 0.02
5.40	143.6	48.4 ± 1.4	49.7	170.1	206,944	0.89	1.60 ± 0.12	0.68 ± 0.02
2.70	149.9	46.7 ± 1.2	48.2	165.6	211,245	0.87	1.64 ± 0.01	0.80 ± 0.04
1.50	155.4	47.2 ± 0.6	48.1	166.6	220,516	0.83	1.43 ± 0.01	0.64 ± 0.02
1.35	134.9	44.7 ± 3.5	46.6	158.1	193,604	0.95	1.44 ± 0.01	0.73 ± 0.03
1.00	144.7	45.1 ± 2.0	46.8	159.5	207,955	0.88	1.32 ± 0.01	0.54 ± 0.06

**BSA**	66.4*^e^*							
1.00	66.5	29.1 ± 0.9	28.8	81	102,486	1.08	NA*^f^*	NA

*^a^* Calculated by the method of Fischer *et al.* (55).

*^b^* Calculated by the method of Rambo *et al.* (45), based on a monomeric solution.

*^c^* Average of χ values determined for the 10 models calculated by the DAMMIF *ab initio* modelling procedure (35).

*^d^* Normalized spatial discrepancies averaged for the 10 models calculated by the DAMMIF *ab initio* modeling procedure (36).

*^e^* Theoretical molecular masses calculated by primary sequence (20).

*^f^* NA, not applicable.

Given these parameters, we approached determination of the full-length protein conformation by analysis of the SAXS data. Ten models were generated for each concentration of Hyl using the *ab initio* bead-modeling program DAMMIF. The averaged χ_(DAMMIF)_ values for each ensemble of 10 models ranged from 1.3 to 1.9 with normalized spatial discrepancies between 0.5 and 0.8 (Table 6). The overall shape is an “L”, with the lower limb of the L representing a larger lobe of the molecule (Fig. 3*C*).

To generate pseudoatomic modules using the SAXS data with our high resolution SpCBM70 structure and existing structures of the PL8 domain, we had to complete the structural coverage of the modules in Hyl because a structure of the ∼70-amino acid linker module has not been determined. This module displays 40% amino acid sequence identity with the analogous module in the structure of the *S. agalactiae* Hyl homolog, allowing us to model the linker module using Phyre2 (44) with the linker module of *S. agalactiae* Hyl as a template (Fig. 3*D*). This resulted in a small compact structure with an Ig-like fold (Fig. 3*E*). Using the structures of the three modules/domains, we used both manual rigid body modeling by fitting the structures into the *ab initio* envelopes (Fig. 3*F*) and automated rigid body modeling using the program BUNCH (not shown) to generate pseudoatomic models. Both approaches failed to achieve χ values better than ∼5–6, thus indicating unsatisfactory models. Plots of *q*^4^ *versus* I(*q*)·*q*^4^ do not plateau in the Porod-Debye region (Fig. 3*G*), suggesting potential conformational heterogeneity (45), leading us to attempt to model the SAXS data using the ensemble optimization method (results not shown); however, this failed to significantly reduce the χ values.

In the absence of any evidence of protein aggregation, the disagreement between the rigid body models and the scattering data suggested that the Hyl protein was present in solution as a mixture of oligomeric states. This was supported by several observations in the DLS and SAXS data. Both the DLS and SAXS data gave similar molecular masses that were ∼30–35% higher than the expected molecular mass and thus outside of an acceptable 10% error limit. The particle densities determined from the SAXS data were also lower than anticipated for an average globular protein, as exemplified by our measurement of ∼1.1 g/cm^3^ for BSA, indicating that the measured Porod volume of the particles in solution is significantly larger than expected for a protein of 110.6 kDa. Finally, the *R_g_* values determined by SAXS appeared to increase with protein concentration. These observations are most consistent with Hyl existing in a monomer/dimer equilibrium, with the presence of the dimer species driving up the apparent molecular mass, decreasing the apparent particle density, and altering the apparent *R_g_* as the equilibrium shifts in response to protein concentration.

To take into account the potential monomer/dimer equilibrium of Hyl in solution and provide insight into the conformation of the protein, we used the program SASREF7MX, which combines rigid body modeling using the composite domains of a protein with modeling mixtures of oligomeric states. In this approach, we used SASREF7MX to model Hyl by treating it as an equilibrium between monomeric and dimeric states, where the conformation of the monomer is identical to that of the monomers comprising the dimer and where the dimer has P2 symmetry. The polypeptide itself was modeled as three separate rigid bodies: SpCBM70, the linker module, and the PL8 domain. Thus, all polypeptide conformations from a single SASREF7MX run, whether they are in the monomeric or dimeric species, are identical reconstructions, yielding a single model of a monomer, a single model of a dimer of these monomers, a measure of the fraction of associated volume for this dimer, and a χ value representing the quality of the model. Because this approach can sometimes result in ambiguous reconstructions, we utilized the maximum distance linking the modules/domains as constraints (46). To further enable our ability to recognize potentially ambiguous or outlying reconstructions, we also ran SASREF7MX 30 times for each concentration of Hyl from 1.0 to 2.7 mg/ml. Each resulting model was separately analyzed with the program OLIGOMER to independently assess the fractions of associated volumes and χ values.

Overall, the χ values from SASREF7MX and OLIGOMER for this pool of 120 models varied from 1.3 to 3.1, depending on the protein sample, and thus the results were substantially improved by considering Hyl as a mixture of oligomers in solution (Table 7). The volumes of the associated fractions determined by SASREF7MX and OLIGOMER were in good agreement and ranged from ∼30 to 40% (Table 7). Consistent with this, an analysis of the monomer/dimer fraction in the DLS data using typical expected radii for a globular monomer of 110.6 kDa (44-Å radius) and for the dimer of 221.2 kDa (60-Å radius) gave dimer fractions of ∼35% (Table 5). Based on conversion of the monomer/dimer fractions to equilibrium concentrations of monomer and dimer for Hyl in solution, and using the general equilibrium equation *K_d_* = [monomer]^2^/[dimer], this equates to *K_d_* values of ∼43 μm from the SAXS data and ∼37 μm from the DLS data.

**TABLE 7:** **Summary of SAXS-based monomer-dimer modeling of Hyl** Values represent the averages and S.D. of 30 independent SASREF7MX runs.

Concentration	SASREF7MX			Oligomer		
	Average χ	Best χ	Average associated fraction	Average χ	Best χ	Average associated fraction
*mg/ml*			%			%
2.70	1.37 ± 0.05	1.31	33.2 ± 1.4	2.21 ± 0.57	1.59	36.5 ± 1.2
1.50	1.33 ± 0.02	1.32	35.9 ± 0.8	1.62 ± 0.19	1.47	39.9 ± 0.8
1.35	1.42 ± 0.04	1.39	26.5 ± 1.7	2.34 ± 0.47	2.25	27.9 ± 5.4
1.00	1.26 ± 0.01	1.25	29.8 ± 1.4	1.40 ± 0.13	1.31	33.1 ± 1.3

**FIGURE 4.**
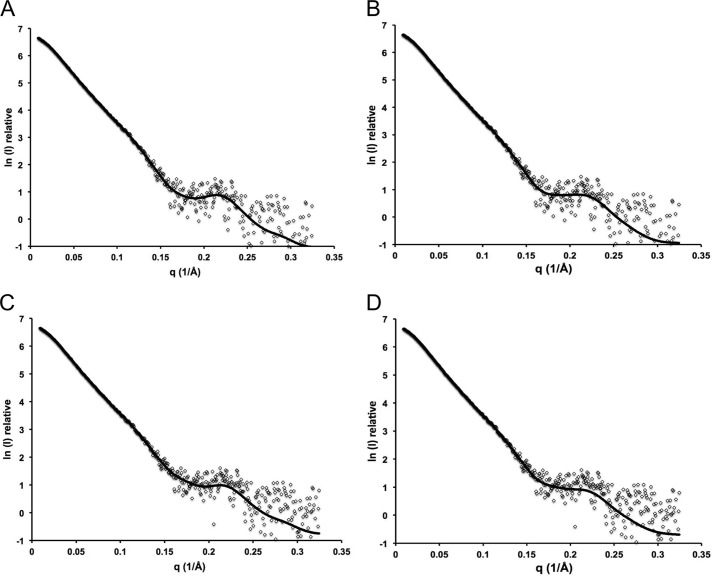
**SASREFMX and OLIGOMER fits of monomer dimer populations.** *A*, SASREF7MX fit for best model of population A; *B*, OLIGOMER fit for best model of population A; *C*, SASREF7MX fit for best model of population B; *D*, OLIGOMER fit for best model of population B. Models were generated with SASREF7MX for Hyl at 1.00 mg/ml.

**FIGURE 5.**
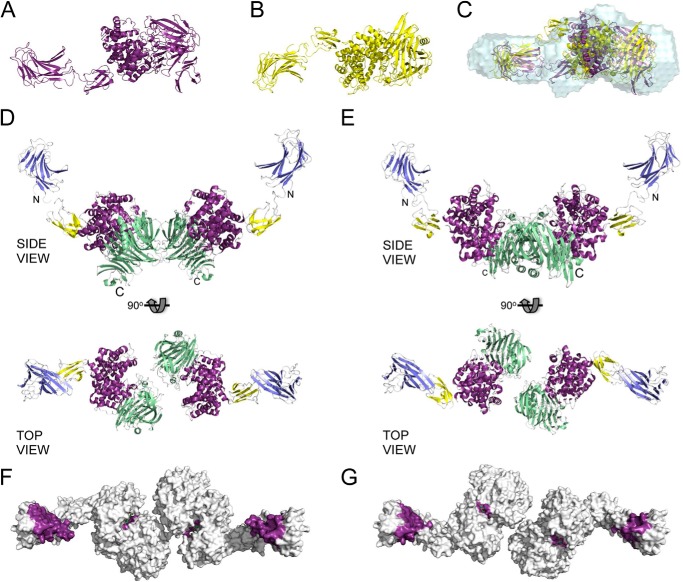
**SAXS-based rigid body modeling of Hyl as an oligomeric mixture.** *A*, model of the Hyl monomer present in population A generated by SASREF7MX rigid body modeling of Hyl as a three-module protein in a monomer/dimer equilibrium. *B*, as in *A* but the Hyl monomer present in population B. *C*, monomers of population A (*purple*) and population B (*yellow*) overlapped with the DAMMIF-generated envelopes of Hyl (1.0 mg/ml) using SUPCOMB. The monomers in *A* and *B* are shown in the same orientation as they are in the *overlapped view* in *C. D* and *E*, dimer structures of populations A and B, respectively, shown from similar *side* and *top views*. The modules are *colored* as follows: CBM (*blue*), linker (*yellow*), α-catalytic domain (*purple*), and β-catalytic domain (*green*). The N and C termini are labeled for reference. *F* and *G*, *top views* of the dimer structures of populations A and B, respectively, as solvent-accessible surface areas with the binding sites of the CBMs *colored purple* and the active site of the PL8 domain *colored purple* and with a bound substrate (*green sticks*; modeled from the structure of the PL8 domain in complex with a 4-mer fragment of hyaluronan; PDB entry 1LXK).

Of the 120 total models, 40 were rejected as likely to be ambiguous reconstructions on the basis of having outlying χ values (>2.0 from SASREF7MX) relative to the best obtained χ values. Notably, the 40 rejected models displayed quite random dimer organizations and monomer conformations (not shown). In contrast, the resulting 80 models clearly divided into two populations, revealing two discreet minima in the modeling process that were reproducible over all of the protein concentrations analyzed. Within a population, the dimer arrangement and conformation of the monomers comprising the dimers were not significantly different, whereas the overall χ values for a single model were excellent, suggesting that the population actually represents a single predominant species that does not display overt flexibility. A comparison between the populations revealed a different dimer arrangement and subtle differences in the monomer conformation. The two populations occurred at roughly equal frequency in the 80 models. On the basis of these results, we propose that each population resulting from our multiple SASREF7MX analyses represents a single possible Hyl monomer and corresponding dimer of the monomers in solution. To represent the best two possible monomer/dimer models, we chose one model from each population giving the best SASREF7MX and OLIGOMER fits to the experimental data (χ values of ∼1.3; Fig. 4) and will refer to them as models A and B. An examination of the monomers in models A and B shows their similar overall extended shapes (Fig. 5, *A* and *B*). Although the *ab initio* DAMMIF models do not represent the mixture of Hyl species present in solution, they do approximate the predominating monomeric species. A comparison of the DAMMIF model with the A and B monomers revealed them to agree well with the *ab initio* shapes; however, the A monomer gave a visually better fit because the “kink” in the *ab initio* shape fits the position of the linker module in this monomer but does not for the B monomer (Fig. 5*C*).

A comparison of A and B dimers shows they share the splayed CBM70 linker arms and that they associate at the PL8 domain (Fig. 5, *D* and *E*). To provide additional general support for the dimerization of Hyl at its PL8 domain, we also used DLS to study the solution properties of the isolated PL8 domain (Table 5), our construct of which was based on the reported domain boundaries used for structural studies of this polypeptide (11, 12). At all of the analyzed concentrations of PL8, the samples showed low polydispersity and thus an absence of significant aggregation. The apparent molecular mass was substantially higher than the expected 82 kDa for the recombinant protein, and both this parameter and the measured radius showed a strong dependence on the protein concentration. These features are consistent with a monomer/dimer equilibrium with an associated fraction similar to that calculated for Hyl by both DLS and SAXS (Table 5). The *K_d_* of dimerization calculated based on estimated equilibrium concentrations is ∼180 μm. The propensity for the PL8 domain to dimerize is less than that for Hyl, which may be due to the truncation of PL8, but, overall, does provide support to the model that Hyl dimerizes via association of the PL8 domain.

**FIGURE 6.**
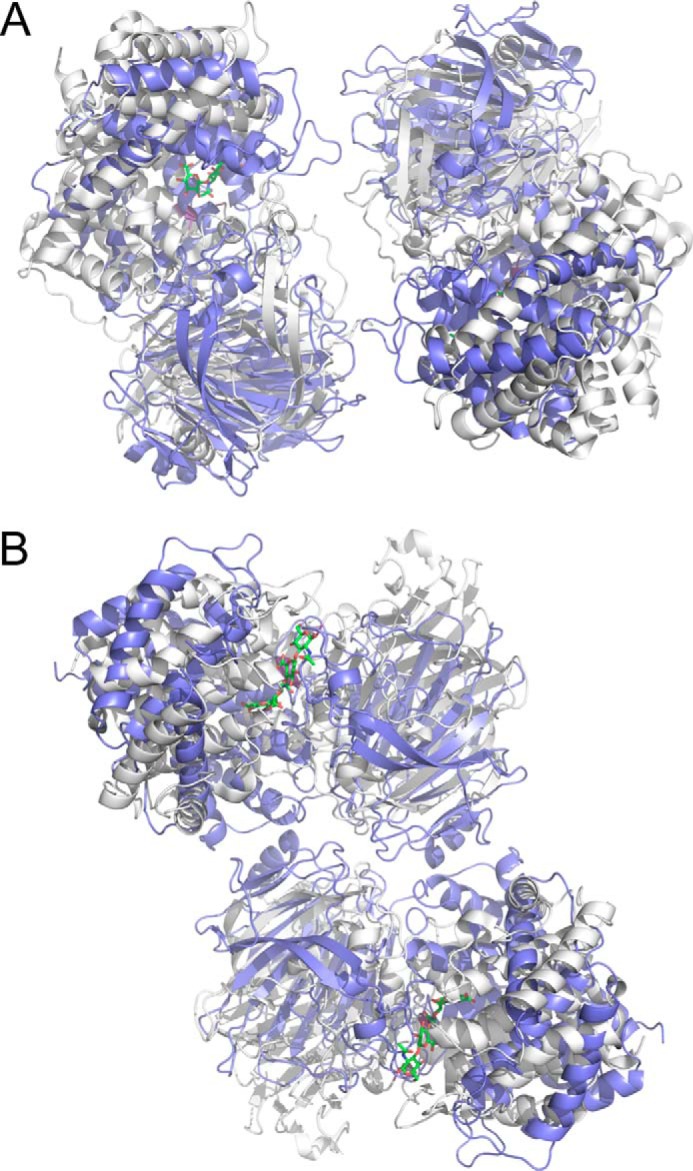
**Comparison of the SAXS-based solution dimers of Hys (CBM and linker not shown for clarity) with dimers of the PL8 domain generated by crystallographic packing in the C2 crystal form (PDB entry 1BRV).** *A*, the dimer of the PL8 domain in population A (*gray*) overlapped with one crystallographic dimer of the PL8 domain (*blue*). *B*, the dimer of the PL8 domain in population B (*gray*) overlapped with the alternate crystallographic dimer of the PL8 domain (*blue*).

Overall, like the monomers, the dimers share highly similar overall shapes, probably explaining why the rigid body reconstructions converged on both models with equal frequency. In the A and B dimers, the differing orientations of the PL8 domains in the monomers result in the generation of different dimerization interfaces in the PL8 domain. For more detailed potential insight into the most likely dimerization interface, we examined the crystal packing of the PL8 domain in the several space groups for which a structure has been solved. A comparison of the PL8 domain crystal packing in space group C2 (PDB entry 2BRV) with the potential A and B dimers shows the major interfaces in the crystal packing to roughly represent both observed dimerization interfaces in the SAXS structures (Fig. 6). This suggests that either dimerization interface in the SAXS structure is possible, because there does indeed appear to be a propensity for these sites on the protein to interact. However, it is unlikely that both possible dimers form in solution. Although we are unable to discriminate conclusively between the two possible dimers, we favor the A dimer as the most plausible on the basis of the observation that the A monomer displays a substantially better match to the *ab initio* reconstructions of Hyl with DAMMIF. Nevertheless, although the arrangements of the core PL8 domain differ in the A and B dimers, the catalytic sites are oriented in roughly the same overall positions in space, whereas the hyaluronan binding sites of the CBM are positioned almost identically (Figs. 4*G* and 5*F*). This indicates that although we cannot conclusively discriminate between the two dimer possibilities, the interpretation of function based on these models is the same overall.

## DISCUSSION

The surface of *S. pneumoniae* is a complex landscape of macromolecules that, in many cases, help to mediate the interaction of the bacterium with the host. Among these molecules involved in the bacterium-host interaction are a wide variety of proteins that are covalently and non-covalently attached to the bacterial surface (47, 48). Carbohydrate-active enzymes are well represented among these proteins and are often associated with pneumococcal virulence, as is the case with Hyl. A common property of the surface-attached pneumococcal enzymes is their multimodularity, but the structures and functions of the modules comprising these important enzymes are not well studied. Of these enzymes, at present, only the surface-attached enzyme SpuA and the secreted family 98 blood group antigen-specific glycoside hydrolases (GH98) have been conclusively demonstrated to have CBMs (49–51). In SpuA, one of the CBM41 modules closes over the active site, aiding directly in substrate recognition, whereas a second module is oriented away from the active site and cell surface to presumably function in adherence to glycogen (52). In the GH98 enzymes, the CBMs are oriented away from the active site and probably function to target these soluble enzymes to glycans on host-cell surfaces (51).

**FIGURE 7.**
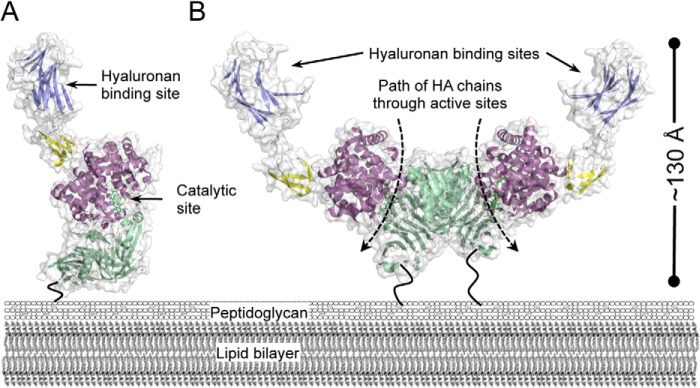
**Modeled surface presentation of Hyl.** Models of the Hyl monomer (*A*) and dimer (*B*) as they may be presented on the surface of the bacterium. Only the structures from population A of the SAXS-generated modules are shown because the two populations are very similar in overall organization. The *dashed arrows* represent the path of processive cleavage of hyaluronan (*HA*) chains through the PL8 domain active sites. The *dark*, *bending lines* represent the unmodeled C-terminal portions of Hyl that tether the protein to the peptidoglycan through a sortase-mediated process.

Here we have shown that Hyl also possesses a CBM, now classified into CBM family 70, that is hyaluronan-specific. Based on our SAXS analysis of Hyl, we propose two possible modes of surface presentation of this enzyme: as a monomer or as a dimer (Fig. 7). In both cases, the presentation is constrained by the orientation of the C-terminal bacterial surface attachment point (LP*X*TG motif), resulting in the CBM probably being most distal from the bacterial surface with its binding site oriented out. The catalytic site would also be generally oriented away from the bacterial surface. Unlike with SpuA, there is no evidence to suggest that that CBM and the catalytic site of Hyl directly cooperate in substrate recognition. However, the co-orientation of the functional sites in the enzyme is consistent with the surface presentation of the enzyme and its role in a partitioned system of two interacting media: the bacterial cell surface and extracellular matrix. This is clearly different from the CBM presentation in the GH98 enzymes, where their soluble deployment appears to allow somewhat random orientation of the CBM binding sites relative to the catalytic sites.

A remarkable feature and novel observation of Hyl is its potential to dimerize. We acknowledge that there is presently a lack of evidence that Hyl is present on the surface of the bacterium as a dimer. However, we have shown that purified Hyl has a relatively strong propensity to dimerize in solution. This dimerization occurs in such a way that the C termini are ideally co-oriented to attach the dimer to the bacterial cell surface while leaving the functional sites in both monomers positioned away from the bacterial surface. Furthermore, the conditions where monomers are being oriented for attachment to the bacterial surface during export could aid in driving dimer formation by reducing the entropic costs of self-association. Thus, the circumstances would seem to favor the presentation of Hyl as dimers on the bacterial surface. Alternatively, Hyl is also found as a soluble protein, and our evidence is in favor of its dimerization under these conditions. In either case (dimerization on the surface or in solution), the dimer adopts a “crablike” shape with functional implications (Fig. 7).

Consistent with most CBM-carbohydrate interactions, the affinity of SpCBM70 for hyaluronan was somewhat modest, with a *K_a_* of ∼1 × 10^5^ m^−1^ (*K_d_* ∼10 μm). Clustering and co-orientation of the CBMs by enzyme dimerization, on the bacterial surface or in solution, would probably promote avidity effects in recognizing hyaluronan aggregated in the extracellular matrix. Indeed, the Lewis^Y^ antigen active GH98 from *S. pneumoniae* has three family 47 CBMs with inherently low individual affinities; however, their tandem presentation takes advantage of avidity to effect an overall increase in apparent affinity (50). The dimerization of a CBM-containing enzyme is an elegant potential solution to exploiting CBM avidity.

Hyl is a processive enzyme first making an endolytic cut and then processing toward the non-reducing end and releasing a disaccharide product with a 4,5-unsaturated non-reducing end d-glucuronic acid. Strikingly, the polarity of the PL8 domain catalytic sites in the dimer of population A (Fig. 5, *B* and *F*) is such that the reducing end of a hyaluronan chain would enter from the top of the dimer (on the CBM side), and the product would be released from the bottom of the dimer as the polymer feeds through the active site in a processive fashion (Fig. 7*B*). In the case of a cell surface-attached dimer, this evokes a model whereby the CBM arms grasp hyaluronan in the extracellular matrix, adhering the bacterium to the hyaluronan, the chains are fed through the PL8 active sites, and then disaccharide product is released right at the bacterial surface. This could optimize transport of the product by a membrane-embedded PTS transporter that is specific for the degradation products of Hyl (10). Alternatively, for soluble Hyl dimer, the conformation is suggestive of a similar model, but the disaccharide products would be released away from the area of binding and degradation on the extracellular matrix, thus potentially making the soluble product more accessible to the bacterium for uptake.

As hypothesized by Rigden and Jedrzejas (53), we have shown that pneumococcal Hyl has a highly specific hyaluronan-binding CBM. Through SAXS analysis of this multimodular enzyme, we have provided evidence that this protein adopts a conformation and quaternary structure that appear to be optimized for cell surface presentation and coordination of hyaluronan binding by the CBM with degradation by the PL8 catalytic module. Overall, these results enhance our understanding of the possible surface presentation and role of such architecturally complex and multifunctional enzymes in the host-pathogen interaction.

## Footnotes

5 The abbreviations used are:

PL8: polysaccharide lyase family 8 (as per CAZy classification)
CBM: carbohydrate-binding module
DLS: dynamic light scattering
GAG: glycosaminoglycan
GH98: glycoside hydrolase family 98, glycogen-degrading endo-β-galactosidase
Hyl: hyaluronate lyase
ITC: isothermal titration calorimetry
NGL: neoglycolipid
SAXS: small angle x-ray scattering
SSRL: Stanford Synchrotron Radiation Lightsource
*D*_max_: maximum particle size
*R_g_*: radii of gyration.
